# Contribution of social factors to readmissions within 30 days after hospitalization for COPD exacerbation

**DOI:** 10.1186/s12890-020-1136-8

**Published:** 2020-04-29

**Authors:** Tadahiro Goto, Kazuki Yoshida, Mohammad Kamal Faridi, Carlos A. Camargo, Kohei Hasegawa

**Affiliations:** 1 Department of Emergency Medicine, Massachusetts General Hospital, 125 Nashua Street, Suite 920, Boston, MA 02114-1101 USA; 2 Department of Epidemiology, Harvard T.H. Chan School of Public Health, Boston, MA USA; 3 Department of Biostatistics, Harvard T.H. Chan School of Public Health, Boston, MA USA; 4 Harvard Medical School, Boston, MA USA

**Keywords:** COPD, Readmission, Socioeconomic status, Acute exacerbation of COPD, Hospitalization

## Abstract

**Background:**

To investigate whether, in patients hospitalized for COPD, the addition of social factors improves the predictive ability for the risk of overall 30-day readmissions, early readmissions (within 7 days after discharge), and late readmissions (8–30 days after discharge).

**Methods:**

Patients (aged ≥40 years) hospitalized for COPD were identified in the Medicare Current Beneficiary Survey from 2006 through 2012. With the use of 1000 bootstrap resampling from the original cohort (training-set), two prediction models were derived: 1) the reference model including age, comorbidities, and mechanical ventilation use, and 2) the optimized model including social factors (e.g., educational level, marital status) in addition to the covariates in the reference model. Prediction performance was examined separately for 30-day, early, and late readmissions.

**Results:**

Following 905 index hospitalizations for COPD, 18.5% were readmitted within 30 days. In the test-set, for overall 30-day readmissions, the discrimination ability between reference and optimized models did not change materially (C-statistic, 0.57 vs. 0.58). By contrast, for early readmissions, the optimized model had significantly improved discrimination (C-statistic, 0.57 vs. 0.63; integrated discrimination improvement [IDI], 0.018 [95%CI, 0.003–0.032]) and reclassification (continuous net reclassification index [NRI], 0.298 [95%CI 0.060–0.537]). Likewise, for late readmissions, the optimized model also had significantly improved discrimination (C-statistic, 0.65 vs. 0.68; IDI, 0.026 [95%CI 0.009–0.042]) and reclassification (continuous NRI, 0.243 [95%CI 0.028–0.459]).

**Conclusions:**

In a nationally-representative sample of Medicare beneficiaries hospitalized for COPD, we found that the addition of social factors improved the predictive ability for readmissions when early and late readmissions were examined separately.

## Background

Chronic obstructive pulmonary disease (COPD) is a major public health problem [1, 2]. In the US, there are approximately 700,000 hospitalizations each year [3] with one-fifth resulting in readmission within 30 days [4]. To curb the healthcare burden, the Hospital Readmissions Reduction Program (HRRP) has started penalizing hospitals for higher than expected rate of 30-day readmission after COPD hospitalization [5]. In addition to hospital-level quality improvement efforts, identification of patients at high risk for readmission and the development of interventions (e.g., care transition interventions) are of great interest to many stakeholders [6–9].

As with claim-based models to predict 30-day readmission after hospitalization for other HRRP-targeted conditions (e.g., heart failure) [10–13], several studies have identified predictors and developed prediction models for readmissions in patients hospitalized for COPD [8, 9, 14–16]. These models incorporated the basic demographics (e.g., age, sex), comorbidities, and in-hospital management (e.g., medication use), with reporting C-statistics of 0.63 to 0.72 [14, 16]. However, these prediction models have been criticized for their lack of detailed social factors (e.g., educational level, marital status) [13, 17–19], and for the assumption that 30-day readmission is a homogeneous process [17, 18, 20]. Indeed, the effects of inpatient management on the readmission risk diminishes rapidly after discharge, reaching a nadir at post-discharge day 7 [18]. Despite the emerging evidence suggesting the involvement of non-clinical factors – such as social factors – in readmission processes [21–23], little is known about whether these factors improve prediction ability and how their contribution varies by timing after COPD hospitalization. In addition, while several studies built prediction models using administrative datasets (e.g., Nationwide Readmission Database [NRD]), these datasets do not include the information on detailed social factors [9, 13, 17–20].

To address this knowledge gap, we used nationally-representative sample of Medicare beneficiaries to test the hypothesis that the addition of social factors to prediction models quantitively improves the predictive ability for 30-day readmission risks in patients hospitalized for COPD. We also examined separately the predictive ability for early readmissions (within 7 days after discharge) and late readmissions (8–30 days after discharge).

## Methods

### Study design and setting

This is a retrospective cohort study of adults hospitalized for COPD using the Medicare Current Beneficiary Survey (MCBS) Access to Care Files from January 2006 through December 2012, provided by the Centres for Medicare & Medicaid Services (CMS) [24]. In brief, the MCBS is a panel survey of a nationally representative sample of Medicare beneficiaries, supplemented with Medicare enrolment and claims data. Each year, approximately 20,000 beneficiaries are inducted to the MCBS as a panel and they are interviewed over a 2-year period [24]. In contrast to Medicare claims data, the MCBS has advantages that it contains the data on socioeconomic characteristics. The unique MCBS data allowed us to derive prediction models with and without social factors, and to compare their performance on predicting readmissions. Additional details of the MCBS may be found elsewhere [24].

In the current study, to enable follow-up of specific patients, inpatient claims for individual patients were linked with the use of the Health Insurance Claim Number-Beneficiary Identification Code. The beneficiary identification code was then used to link beneficiary enrolment and demographics information from the Medicare Master Beneficiary Summary File. Patient comorbidities are characterized using the *International Classification of Diseases, Ninth Revision, Clinical Modification* (*ICD-9-CM*) diagnosis codes. This study was approved by the institutional review boards of Massachusetts General Hospital with an informed consent waiver due to the retrospective nature of this study. We reported the study according to the TRIPOD (Transparent Reporting of a Multivariable Prediction Model for Individual Prognosis or Diagnosis) statement for reporting multivariable prediction model development and validation [25].

### Study population

We identified all unplanned COPD hospitalizations (index hospitalizations) made by patients aged ≥40 years. We defined the unplanned hospitalization for COPD using *ICD-9-CM* diagnostic codes (*ICD-9-CM* diagnosis codes: 491.21, 491.22, 491.8, 491.9, 492.8, 493.20, 493.21, 493.22, and 496), or those with a primary diagnosis of respiratory failure (*ICD-9-CM* diagnosis codes: 518.81, 518.82, 518.84, and 799.1) and secondary diagnosis of COPD (Table S1) [16]. When a patient met any of the following criteria according to the CMS publicly-reported readmission measures [16], we excluded the patient from the analysis: patients who left the hospital against medical advice, those who were transferred to another acute care facility, those who died during the index hospitalization, those without continuous enrolment in Medicare fee-for-service for 1 year prior to the date of the index hospitalization, and those without at least 30 days post-discharge enrolment in Medicare fee-for-service. When a patient had multiple readmissions after the index hospitalization for COPD, we used only the first readmission within 30 days of discharge as a readmission. Therefore, additional readmissions within the 30-day period were not counted as readmissions or index hospitalizations) [16]. To maintain the consistency with the CMS publicly-reported readmission measures, we considered subsequent hospitalizations occurring after 30 days from discharge as index hospitalizations if they met the inclusion criteria [16].

### Outcome measures

The outcome measure was a readmission at any hospital for any reason occurring within 30 days of discharge from the index hospitalization for COPD, according to the CMS definition [16]. Early readmissions (within 7 days after discharge) and late readmissions (8–30 days after discharge) were also examined separately [18, 26–28]. In the primary analysis for late readmissions, we excluded patients who had an early readmission because these patients did not have a late readmission as we considered only the first readmission within 30 days of discharge as a readmission. We consolidated the principal discharge diagnoses (> 14,000 *ICD-9-CM* diagnosis codes) at the readmission into 285 mutually exclusive diagnostic categories using the Agency for Healthcare Research Quality’s Clinical Classifications Software (CCS) [29], according to previous studies [30–32].

### Candidate predictor variables and model derivation

To derive the prediction models with and without social factors, we generated 1000 bootstrap samples (*n* = 905) from the original cohort (training set) and fitted two logistic regression models. The two prediction models were 1) the reference model based on the CMS readmission measurements [16] and 2) the optimized model including social factors in addition to the variables used in the reference model. In the bootstrapping, the current study created 1000 bootstrap samples (each bootstrap sample has *n* = 905) that are consisted of subsamples from the original 905 patients *with* replacement. In each bootstrap sample, we derived a model to estimate regression coefficients. We have repeated this procedure 1000 times (for each of 1000 bootstrap samples), and averaged the regression coefficients across the 1000 models. These averaged regression coefficients were then used to develop the final prediction models. In the reference model, according to the CMS readmission measurements, we included patient’s age, comorbidities, and mechanical ventilation use (Table S2). Because of the limited sample size of the study (*n* = 905; readmissions = 167), we grouped 38 comorbidities into 10 categories according to the organ system (i.e., cardiac, central nervous system, endocrine, gastrointestinal, hematologic, musculoskeletal, neoplasms, psychiatric, respiratory, and others; Table S2). Respiratory comorbidities include sleep apnea, respiratory arrest, cardio-respiratory failure and shock, fibrosis of lung and other chronic lung disorder, and pneumonia [16].

In the optimized model, based on a priori knowledge and clinical plausibility [33–38], we included social factor variables that are available in the MCBS, in addition to the variables used in the reference model above (i.e., the optimized model included patient’s age, comorbidities, mechanical ventilation use, and all of the following variables). As the use of arbitrary statistical cutoffs for variable selection (e.g., univariate screening) has been criticized [39, 40], we have selected the predictors in the final model based on a priori knowledge. The included social factors were educational level (8th grade or less, some high school education, high school graduate, and others), marital status (current spouse), number of children (0, 1, and ≥ 2), limitations on activities and social life, poverty status (annual household income less than $25,000), and patient residence (urban vs. rural). The poverty threshold of <$25,000 was used based on the *Supplemental Poverty Measure* published by the U.S. Census Bureau [41]. The urban–rural status of the patient residence was defined according to the National Center for Health Statistics guidelines [42]. We have ensured no multicollinearity by calculating variance inflation factors (all, < 1.5). Additionally, to examine whether social factors improve prediction ability, we did not add variables other than social factors.”

### Prediction model performance

Next, to examine the performance of each prediction model, we fit the prediction models to the original cohort (test set). We examined the discrimination ability using C-statistic and integrated discrimination improvement (IDI) [43] and compared the reclassification ability using the continuous net reclassification index (NRI) [43]. We also measured the prediction performance of each model by using decision curve analysis. The decision curve analysis is a measure that takes into account the different weights of different misclassification types with a direct clinical interpretation (e.g., trade-offs between under-estimation and over-estimation of the risk of readmission in each model) [44, 45]. Specifically, the relative impact of false-negative (under-estimation of the readmission risk) and false-positive (over-estimation of the readmission risk) results given a threshold probability (or clinical preference) was accounted to yield the net benefit of each model. The net benefit of each model over a specified range of threshold probabilities of outcome is graphically displayed as a decision curve. We used bootstrapped samples to yield bias-corrected (overfitting-corrected) estimates of predicted vs. observed values based on subsetting predictions on nonparametric smoothers by using R *rms* package [46]. For logistic models, a nonparametric calibration curve was estimated over a sequence of predicted values, and error referred to the difference between the predicted values and the corresponding bias-corrected calibrated values. In the sensitivity analysis, for late readmissions, we repeated the analysis including patients with early readmissions (i.e., including patients who already had an outcome event). A two-tailed *P* < 0.05 was considered statistically significant. We analysed data using STATA 15.0 (StataCorp, College Station, TX) and R 3.4.1 (R Foundation). We used *pROC* package for plotting the receiver operator curves [47], and *Hmisc* package for calculating IDI and NRI [48].

## Results

### Characteristics of the cohort

From 2006 to 2012, there were a total of 136,024 subjects recorded in the MCBS data. Of these, 2049 subjects had at least one hospitalization for COPD, corresponding to 1034 hospitalizations for COPD as the primary discharge diagnosis. Among the 1034 hospitalizations, 905 hospitalizations (640 subjects) met the criteria for index hospitalization (28% were made by multiple hospitalizations). At the index hospitalization, the median age was 76 years, 54% were women, and 85% were non-Hispanic white (Table 1). As for the socioeconomic characteristics, approximately 50% had an education level of high school graduate or higher, one-third were currently married, and 90% had ≥1 child. Additionally, approximately two-thirds reported limitations on activities and social life and were living in metropolitan area. Within 30 days after hospital discharge, 18.5% (95%CI, 16.1–21.1%; *n* = 167) of these patients were readmitted; 7.9% (95%CI, 6.4–9.9%; *n* = 72) were readmitted within 7 days after discharge (early readmissions) and 10.5% (95%CI 8.7–12.7%; *n* = 95) were readmitted during 8–30 days after discharge (late readmissions). The most frequent reason of the 30-day readmission was COPD (22%), followed by pneumonia (13%) and congestive heart failure (11%) (Table S3). Respiratory-related readmissions (e.g., acute exacerbation of COPD, pneumonia) accounted for approximately 50% of readmissions.

**Table 1:** Baseline characteristics of the original cohort

	**Overall**	**Readmitted**	**Not readmitted**
**Characteristics**	***n*** **= 905**	***n*** **= 167**	***n*** **= 738**
Age (year) median (interquartile range)	76 (68–82)	74 (68–82)	76 (68–82)
Sex			
Male	415 (46%)	71 (43%)	344 (46%)
Female	490 (54%)	96 (57%)	394 (53%)
Non-Hispanic white race	770 (85%)	152 (20%)	618 (84%)
Smoking status			
Current smoker	194 (21%)	31 (16%)	163 (22%)
Former smoker	524 (58%)	89 (17%)	435 (59%)
Never smoker	187 (21%)	47 (25%)	140 (19%)
Comorbidities*			
Cardiac diseases	561 (62%)	115 (21%)	446 (60%)
Central nervous system diseases	50 (6%)	11 (22%)	39 (5%)
Endocrine diseases	293 (32%)	45 (15%)	248 (34%)
Gastrointestinal diseases	246 (27%)	46 (19%)	200 (27%)
Hematologic diseases	130 (14%)	22 (17%)	108 (15%)
Musculoskeletal diseases	36 (4%)	10 (28%)	26 (4%)
Neoplasms	34 (4%)	3 (9%)	31 (4%)
Psychiatric diseases	270 (30%)	50 (19%)	220 (30%)
Respiratory diseases	275 (30%)	45 (16%)	230 (31%)
Other diseases	52 (6%)	10 (19%)	42 (6%)
Mechanical ventilation use during the index hospitalization	56 (6%)	13 (23%)	43 (6%)
Socioeconomic factors			
Educational level			
8th grade or less	157 (17%)	36 (23%)	121 (16%)
Some high school education	232 (26%)	46 (20%)	186 (25%)
High school graduate	491 (54%)	79 (16%)	412 (56%)
Not responded	25 (3%)	6 (24%)	19 (3%)
Marital status (currently married)	313 (35%)	52 (17%)	261 (35%)
Number of children			
0	99 (11%)	22 (22%)	77 (10%)
1	317 (35%)	23 (17%)	294 (40%)
≥ 2	489 (54%)	122 (18%)	367 (50%)
Limitations on activities and social life	584 (65%)	108 (19%)	476 (64%)
Poverty (annual household income <$25,000)	531 (59%)	103 (19%)	428 (58%)
Living in metropolitan area	564 (62%)	97 (17%)	467 (63%)

* 38 chronic conditions (defined by the Centres for Medicare and Medicaid Services Readmission Measurements; Table S2) categorized into 10 categories according to the organ systems

### Predictive models and their performance on overall 30-day readmissions

**Fig. 1:**
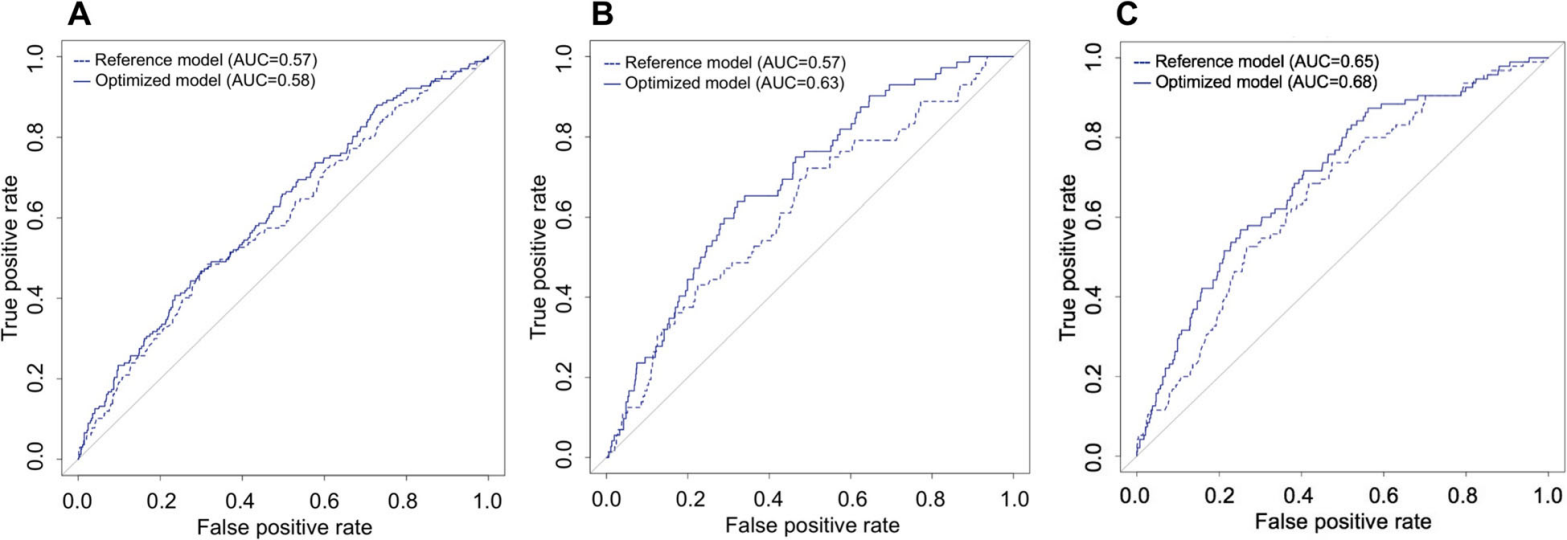
Prediction performance of the reference and optimized models on readmission within 30 days after hospitalization for COPD, according to readmission outcomes. **a** The receiver operating characteristic (ROC) curves for predicting overall 30-day readmissions after hospitalization for COPD. There were no material differences in the area-under-curve (AUC) between reference model (AUC, 0.57) and optimized model (AUC, 0.58). **b** The ROC curves for predicting early readmissions within 7 days after hospitalization for COPD. The addition of social factors to the reference model improved the AUC from 0.57 to 0.63. **c** The ROC curves for predicting late readmissions during 8–30 days after hospitalization for COPD. The addition of social factors to the reference model improved the AUC from 0.65 to 0.68

In the training set, the derived models predicting the overall 30-day readmissions with corresponding odds ratios (ORs) are shown in Table 2. In the reference model, cardiac disease comorbidity was the only significant predictor for 30-day readmission. In the test set, the C-statistic of the derived reference model was 0.57 (95%CI, 0.52–0.62; Fig. 1a).

**Table 2:** Prediction model derivations, according to readmission outcomes in the training set using 1000 bootstrap samples

**Predictors**	**Overall 30-day readmission**		**Early readmission** **(within 7 days after discharge)**		**Late readmission** **(8–30 days after discharge)**	
	**Reference model**^**a**^ **OR (95%CI)**	**Optimized model**^**b**^ **OR (95%CI)**	**Reference model**^**a**^ **OR (95%CI)**	**Optimized model**^**b**^ **OR (95%CI)**	**Reference model**^**a**^ **OR (95%CI)**	**Optimized model**^**b**^ **OR (95%CI)**
Age (continuous variable), year	0.99 (0.98–1.01)	0.99 (0.98–1.01)	1.00 (0.98–1.03)	1.00 (0.98–1.02)	0.99 (0.97–1.01)	0.99 (0.97–1.01)
Respiratory diseases	0.81 (0.55–1.18)	0.80 (0.54–1.16)	1.44 (0.86–2.39)	1.40 (0.83–2.32)	0.48 (0.27–0.83)	0.47 (0.26–0.81)
Cardiac diseases	1.49 (1.03–2.19)	1.52 (1.04–2.24)	0.83 (0.50–1.39)	0.81 (0.48–1.37)	2.39 (1.44–4.13)	2.62 (1.56–4.57)
Neoplasms	0.42 (0.10–1.21)	0.42 (0.10–1.21)	0.70 (0.11–2.40)	0.71 (0.11–2.48)	0.24 (0.01–1.19)	0.25 (0.01–1.23)
Endocrine diseases	0.71 (0.48–1.04)	0.70 (0.47–1.02)	0.77 (0.44–1.31)	0.74 (0.41–1.26)	0.67 (0.40–1.08)	0.69 (0.41–1.13)
Gastrointestinal diseases	1.04 (0.70–1.51)	1.04 (0.70–1.53)	0.86 (0.48–1.49)	0.86 (0.47–1.51)	1.17 (0.71–1.88)	1.12 (0.67–1.84)
Hematologic diseases	0.95 (0.57–1.54)	0.90 (0.53–1.47)	1.02 (0.48–1.98)	0.96 (0.44–1.88)	0.88 (0.44–1.63)	0.89 (0.44–1.67)
Psychiatric diseases	0.99 (0.67–1.43)	0.95 (0.64–1.39)	1.32 (0.77–2.20)	1.17 (0.68–1.99)	0.78 (0.47–1.27)	0.80 (0.48–1.32)
Central nervous system diseases	1.39 (0.66–2.72)	1.44 (0.68–2.85)	1.37 (0.46–3.31)	1.39 (0.46–3.42)	1.31 (0.47–3.06)	1.47 (0.52–3.50)
Musculoskeletal diseases	1.85 (0.83–3.87)	1.80 (0.79–3.79)	1.99 (0.65–4.99)	1.77 (0.57–4.58)	1.68 (0.54–4.35)	1.90 (0.60–5.09)
Other diseases	1.03 (0.47–2.04)	1.09 (0.50–2.20)	1.05 (0.30–2.74)	1.23 (0.35–3.31)	1.01 (0.37–2.33)	0.97 (0.35–2.29)
Mechanical ventilation use	1.38 (0.69–2.59)	1.38 (0.69–2.62)	2.32 (1.01–4.83)	2.29 (0.98–4.88)	0.73 (0.21–1.91)	0.69 (0.20–1.83)
**Socioeconomic factors**						
Educational level						
8th grade or less		1 (reference)		1 (reference)		1 (reference)
Some high school education		0.82 (0.49–1.38)		0.94 (0.45–1.99)		0.66 (0.35–1.25)
High school graduate		0.67 (0.42–1.10)		0.59 (0.30–1.20)		1.22 (0.47–3.00)
Not responded		1.15 (0.38–3.10)		1.03 (0.21–3.72)		0.89 (0.46–1.75)
Marital status (currently married)		0.85 (0.56–1.26)		0.59 (0.32–1.06)		1.22 (0.73–2.03)
Number of children						
0		1 (reference)		1 (reference)		1 (reference)
1		0.72 (0.37–1.42)		1.10 (0.47–2.66)		0.45 (0.16–1.22)
≥ 2		0.81 (0.48–1.41)		0.62 (0.31–1.37)		0.99 (0.49–2.13)
Limitations on activities and social life		1.00 (0.69–1.46)		0.97 (0.58–1.66)		1.03 (0.63–1.70)
Poverty status (Annual household income <$25,000)		0.95 (0.63–1.42)		0.47 (0.26–0.82)		1.78 (1.03–3.13)
Living in metropolitan area		0.79 (0.55–1.13)		0.71 (0.42–1.19)		0.81 (0.51–1.31)

*Abbreviations*: *OR* Odds ratio, *CI* Confidence interval

^a^The reference model includes the variables derived from the Centres for Medicare and Medicaid Services Readmission Measurements

^b^The optimized model includes socioeconomic factors in addition to the variables in the reference model. Details of comorbidities are shown in Table S2

In the optimized model including the social factors, cardiac disease comorbidity was also significant predictor for 30-day readmissions. The C-statistic of the optimized model was 0.58 (95%CI, 0.54–0.63; Fig. 1a). Compared with the reference model, there were no significant differences in discrimination (IDI, 0.009 [95%CI, − 0.001 to 0.020]) and reclassification (NRI, 0.135 [95%CI, − 0.031 to 0.302]) (Table 3). The model calibration curves for each model are shown in Figure S1.

**Table 3:** Prediction model performance in the test set, according to readmission outcomes

**Model performance measures**	**Overall 30-day readmission**		**Early readmission** **(within 7 days after discharge)**		**Late readmission** **(8–30 days after discharge)**	
	**Reference model**	**Optimized model**	**Reference model**	**Optimized model**	**Reference model**	**Optimized model**
C statistic (95%CI)	0.57 (0.52 to 0.62)	0.58 (0.54 to 0.63)	0.57 (0.50 to 0.64)	0.63 (0.56 to 0.70)	0.65 (0.60 to 0.71)	0.68 (0.62 to 0.73)
Integrated discrimination improvement (95%CI)	–	0.009 (−0.001 to 0.020)	–	0.018 (0.003 to 0.032)	–	0.026 (0.009 to 0.042)
Continuous net reclassification improvement, (95%CI)	–	0.135 (− 0.031 to 0.302)	–	0.298 (0.060 to 0.537)	–	0.243 (0.028 to 0.459)

*Abbreviation*: *CI* Confidence interval

Models were derived from 1000 bootstrap resamples

For the model comparisons, the integrated discrimination improvement and net reclassification improvement values of > 0 indicate that, compared to the reference model, the optimized model has better discrimination and reclassification performance

### Predictive models and their performance on early readmissions

In the reference model predicting early readmissions (readmission within 7 days after discharge), the use of mechanical ventilation was the only significant predictor (Table 2). By contrast, in the optimized model, poverty status was significant predictor for lower risks of early readmissions. In the test set, the C-statistic of the derived reference model was 0.57 (95%CI, 0.50–0.64; Fig. 1b) and that of the optimized model was 0.63 (95%CI, 0.56–0.70). The IDI and NRI indicated that, compared to the reference model, the optimized model had significantly higher discrimination (IDI, 0.018 [95%CI, 0.003 to 0.032]) and reclassification (continuous NRI, 0.298 [95%CI, 0.060 to 0.537]) (Table 3). The model calibration curves for each model are shown in Figure S2.

### Predictive models and their performance on late readmissions

After excluding 72 index hospitalizations who had early readmissions, in the both models predicting late readmissions (readmission during 8–30 days after discharge), cardiac disease comorbidity and respiratory comorbidity were significant predictors. Additionally, in the optimized model (Table 2), poverty status was a predictor for higher risks of late readmission. In the test set, the C-statistic of the derived reference model was 0.65 (95%CI, 0.60–0.71; Fig. 1c) and that of the optimized model was 0.68 (95%CI, 0.62–0.73). The IDI and NRI indicated that, compared with the reference model, the optimized model had significantly higher discrimination (IDI, 0.026 [95%CI, 0.009 to 0.042]) and reclassification (continuous NRI, 0.243 [95%CI, 0.028 to 0.459]) (Table 3). The model calibration curves for each model are shown in Figure S3. In the sensitivity analysis including early readmissions, these findings did not change materially (Table S4 and Figure S4).

### Decision curve analysis

In the decision curve analysis, compared with the reference model, the optimized model had a greater net benefit in the 15–20% range of probability of overall 30-day readmission (Figure S5 A). Additionally, the optimized models also had a greater net benefit in predicting both early and late readmissions in a plausible range of readmission probabilities (Figure S5 B and C).

## Discussion

By using nationally-representative sample of US Medicare beneficiaries, we found a potential benefit of adding social factors to the CMS-based reference model to improve the predictive ability for readmission within 30 days after COPD hospitalization. When we examined early and late readmissions separately, the predictive ability of optimized models were also significantly higher than that of the corresponding reference model. The decision curve analysis indicates the greater net benefit of optimized model over the reference model for thresholds between 15 and 20% of probability of 30-day readmission. Additionally, the contribution of predictive factors (e.g., cardiac comorbidity, poverty status) to the readmission risk differed between early and late readmissions. To the best of our knowledge, this is the first study that has investigated the incremental benefit of social factors on predicting the risk of readmissions – including early and late readmissions – in patients hospitalized for COPD. Given that the current one-size-fits-all approach (i.e., HRRP) has not been successful at lowering numbers of 30-day COPD readmissions [49, 50], our findings demonstrating the heterogeneity of the 30-day readmission should help identify patients at high risk for readmission and inform the development of more targeted preventive interventions.

Despite the evidence suggesting the associations between social factors and readmission risks in various disease conditions (e.g., heart failure) [33–37], most studies in the COPD population have focused on patient and hospital factors as predictor for readmission [8, 9, 14–16]. Of these, few studies have used other factors to develop models predicting readmissions [7, 14]. For example, in an US-based study of patients with COPD (age 40–64 years) using a commercial insurance database, Sharif et al. reported that the C-statistic of prediction model for 30-day readmissions improved to 0.72 after adding provider-level (e.g., medication prescriptions) and system-level (e.g., hospital length-of-stay) factors to their reference model that had the C-statistic of 0.68 [14]. Studies using large datasets (e.g., NRD, Medicare data) have shown that some proxy social factors (i.e., quartiles of household income that are estimated by ZIP code, insurance status) were related to COPD readmissions [13, 51]. However, the NRD and Medicare data do not include detailed social factors (e.g., marital status, actual income, number of children). In another single-centre retrospective study of 109 Canadian patients, while the prediction performance was not examined, Wong et al. reported that marital status (single) was a significant predictive factor for readmission following hospitalization for COPD [52]. In non-COPD populations, (e.g., acute myocardial infarction, heart failure, pneumonia), the emerging evidence has suggested the importance of social factors to improve the prediction ability for readmission risks [22, 37, 53, 54]. In addition, an earlier study examined the association of the lower income with the risk of acute exacerbation of COPD in patients aged 40–65 years with COPD [55]. While the previous study had the different design and outcomes (COPD exacerbation vs. 30-day readmission), the importance of social factors (e.g., poverty) in the association of and prediction for the COPD morbidity risk is consistent. Consequently, our study corroborates these prior studies, and extends them by demonstrating, in a nationally-representative sample of Medicare beneficiaries, the incremental benefit of social factors on the CMS models to predict readmissions in patients with COPD.

While we found the additional benefit of social factors on predicting both early and late readmission, our findings also support the heterogeneity of the “30-day readmission” outcome. For example, mechanical ventilation use was a predictor for early readmissions but not for overall 30-day readmissions or late readmissions. By contrast, cardiac comorbidity and poverty status were significant predictors for late readmissions. The relative decrease in the effect of acute clinical factors and in-hospital factors (such as the use of mechanical ventilation) over time is clinically plausible. Indeed, the effects of inpatient care on the risk for readmissions diminish rapidly within a week after discharge [18], with a recovery from symptoms of COPD exacerbation [28]. In contrast, as a patient returns to the community, the relative importance of social factors (e.g., poverty) and chronic conditions (e.g., comorbid cardiac diseases) increases over time. While poverty status was associated with a lower risk of early readmission in this study, it is possible that poverty functioned as a barrier to accessing health care due to the costs of seeking health care, which include not only out-of-pocket spending on care but also transportation costs [56]. Consequently, patients with poverty may have avoided having ambulatory health care and/or presenting to hospitals until the later period, which would reduce the rate of early readmission but increase the rate of late readmission. The latter finding can also be explained by the observations from earlier studies. For example, the literature has suggested that poverty is associated with the lack of health literacy affecting adherence to post-discharge instructions [54, 57]. Without social support, some patients would not be able to cope with the post-hospital syndrome, a transient condition of generalized risk after hospitalization [58, 59]. By contrast, positive social support provided by family members has been associated with improved quality of life [60] and better pulmonary rehabilitation adherence [61], resulting in reduced late readmissions. While marital status and number of children may be indicators for social support, living status (e.g., living alone) has been reported as an important prognostic factor. Indeed, an observational study reported that, patients with COPD who lived alone had lower levels of physical activity and lower rates of pulmonary rehabilitation participation compared with patients who live with other family members [62]. Taken together, hospital readmission is a complex construct involving multiple factors, such as patient factors, inpatient and outpatient care, and social factors. Accordingly, to reduce readmissions and improve outcomes in patients with COPD, it is imperative to developing multifaceted strategies targeting each of underlying constructs – e.g., provision of high quality of inpatient, transition, and outpatient care, improvement in access to ambulatory care after hospital discharge, and social support in the community [63, 64].

### Potential limitations

Our study has several potential limitations. First, as with other studies using claims data, misclassification of hospitalizations is possible. However, the definitions using the CMS publicly-reported readmission measure [11] have a high specificity and positive predictive value (both ≥90%) [65]. Furthermore, the Medicare data are widely used for clinical research because the data are rigorously tested and considered accurate. Second, we did not account for several clinical information including the chronic severity of COPD since the MCBS and Medicare data do not contain such clinical information. Third, while the predictive ability of the optimized model was not high, the study objective was *not* to develop clinical prediction models but, rather, to examine the incremental benefit of social factors on predicting the readmission risk in the population. Fourth, because of the relatively limited sample size and unavailability of unique dataset that has social factors, we used bootstrap samples to develop the model (rather than the use of an external sample). In addition, the limited sample size precluded us from estimating the detailed predictive contribution (e.g., the predictive contribution of specific cancers for the risk of readmission). Yet, formal validation of our study in separate populations is necessary. In addition, the grouping of 38 comorbidities into 10 categories according to the organ system may not yield the same predictive ability with the original model. Fifth, as we excluded patients who left the hospital against medical advice according to the CMS readmission measurement. This might cause selection bias. Lastly, the study sample comprised Medicare beneficiaries, and, therefore, our inference may not be generalizable to non-Medicare individuals with COPD or other non-US settings where social factors and their effects may differ [66]. Nonetheless, our study population accounts for 70% of hospitalizations for COPD in the US [4].

## Conclusions

Based on nationally-representative sample of Medicare beneficiaries hospitalized for COPD, we found that the addition of social factors to the prediction model quantitatively improves the predictive ability for early and late readmissions. We also found that inpatient care factor (e.g., the use of mechanical ventilation) was a predictor for early readmissions while comorbidity and social factors (e.g., poverty) were predictors for late readmissions, suggesting that the readmission is a complex and heterogeneous construct. Despite the modest predictive ability for the clinical use, the improvement of predictive ability has important implications for researchers and policy makers. For researchers, our observations should facilitate further investigations into better identification of patients with COPD at high risk for readmissions. For policy-makers, our findings underscore the importance of continued efforts to develop and implement preventive strategies (e.g., high quality inpatient, transition, and outpatient management, as well as optimization of post-discharge environment) to reduce readmissions in this high-risk population.

## Supplementary information

**Additional file 1.**

## Data Availability

Medicare Current Beneficiary Survey data can be purchased from the Centres for Medicare and Medicaid Services.

## Abbreviations

AECOPD: acute exacerbation of chronic obstructive pulmonary disease
CMS: Centers for Medicare and Medicaid Services
COPD: chronic obstructive pulmonary disease
HRRP: Hospital Readmission Reduction Program
ICD-9-CM: *International Classification of Diseases, Ninth Revision, Clinical Modification*
MCBS: Medicare Current Beneficiary Survey
