# Supplementary material for: Contribution of social factors to readmissions within 30 days after hospitalization for COPD exacerbation
Source: BMC Pulm Med. 2020 Apr 29;20:107. doi: 10.1186/s12890-020-1136-8 (PMC7191726; doi:10.1186/s12890-020-1136-8)
Supplement: Supplementary file 1 — Additional file 1. [file 12890_2020_1136_MOESM1_ESM.doc]

**SUPPLEMENTARY INFORMATION**

**TITLE: Contribution of social factors to readmissions within 30 days after hospitalization for COPD exacerbation**

**Authors’ full names:** Tadahiro Goto, MD, MPH^1^; Kazuki Yoshida, MD, MPH^2,3^; Mohammad Kamal Faridi, MPH^1^; Carlos A. Camargo, Jr. MD, DrPH^1,2,4^; and Kohei Hasegawa, MD, MPH^1,4^

**Authors’ affiliation(s):**

1. Department of Emergency Medicine, Massachusetts General Hospital, Boston, MA
2. Department of Epidemiology, Harvard T.H. Chan School of Public Health, Boston, MA
3. Department of Biostatistics, Harvard T.H. Chan School of Public Health, Boston, MA
4. Harvard Medical School, Boston, MA

**Figure S1***-* **Model calibration curves of the prediction models for readmission within 30 days after hospitalization for COPD**


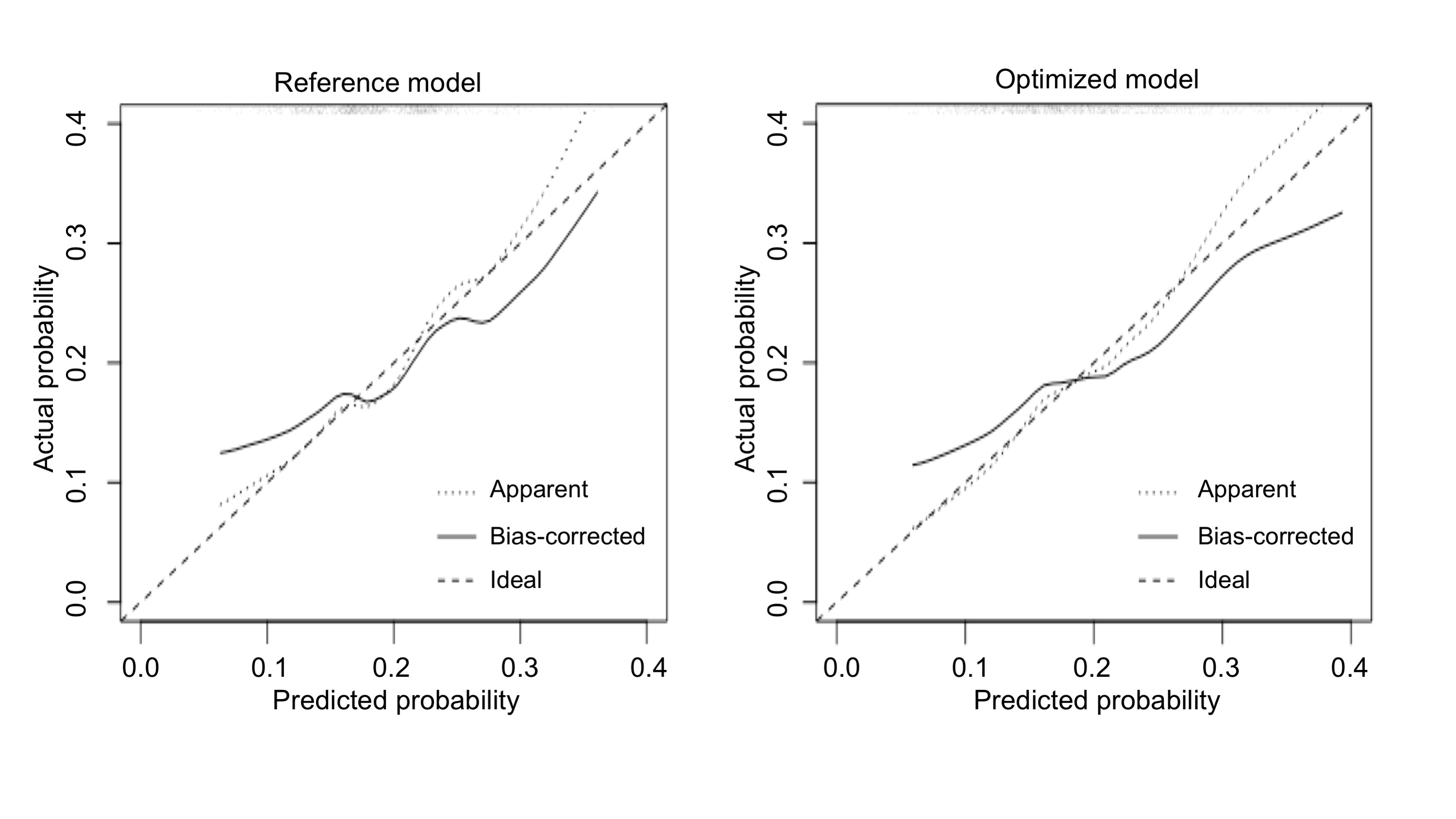


**Figure S2- Model calibration curves of the prediction models for readmission within 7 days after hospitalization for COPD**

**
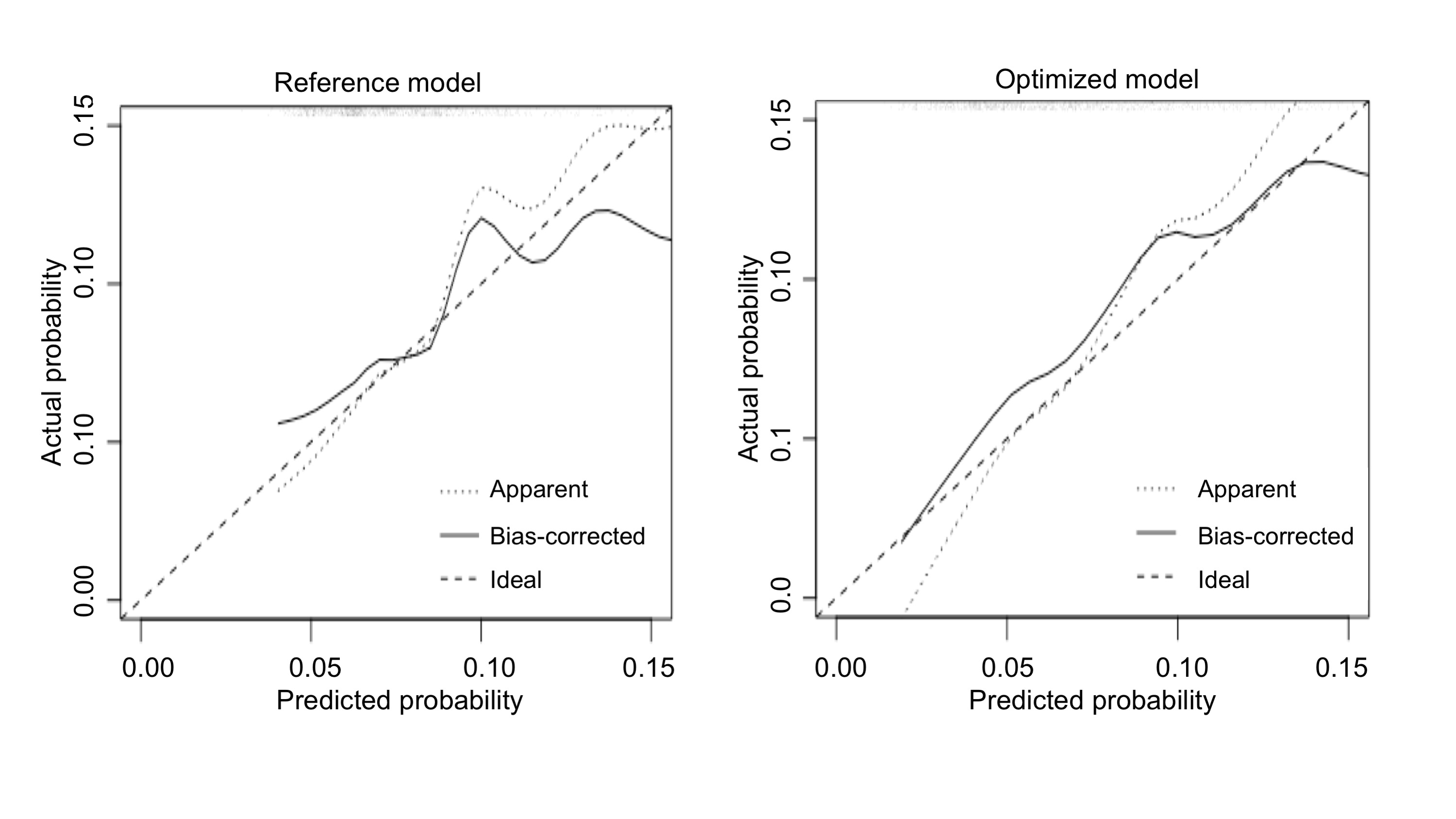
**

**Figure S3- Model calibration curves of the prediction models for readmission 8-30 days after hospitalization for COPD**

**
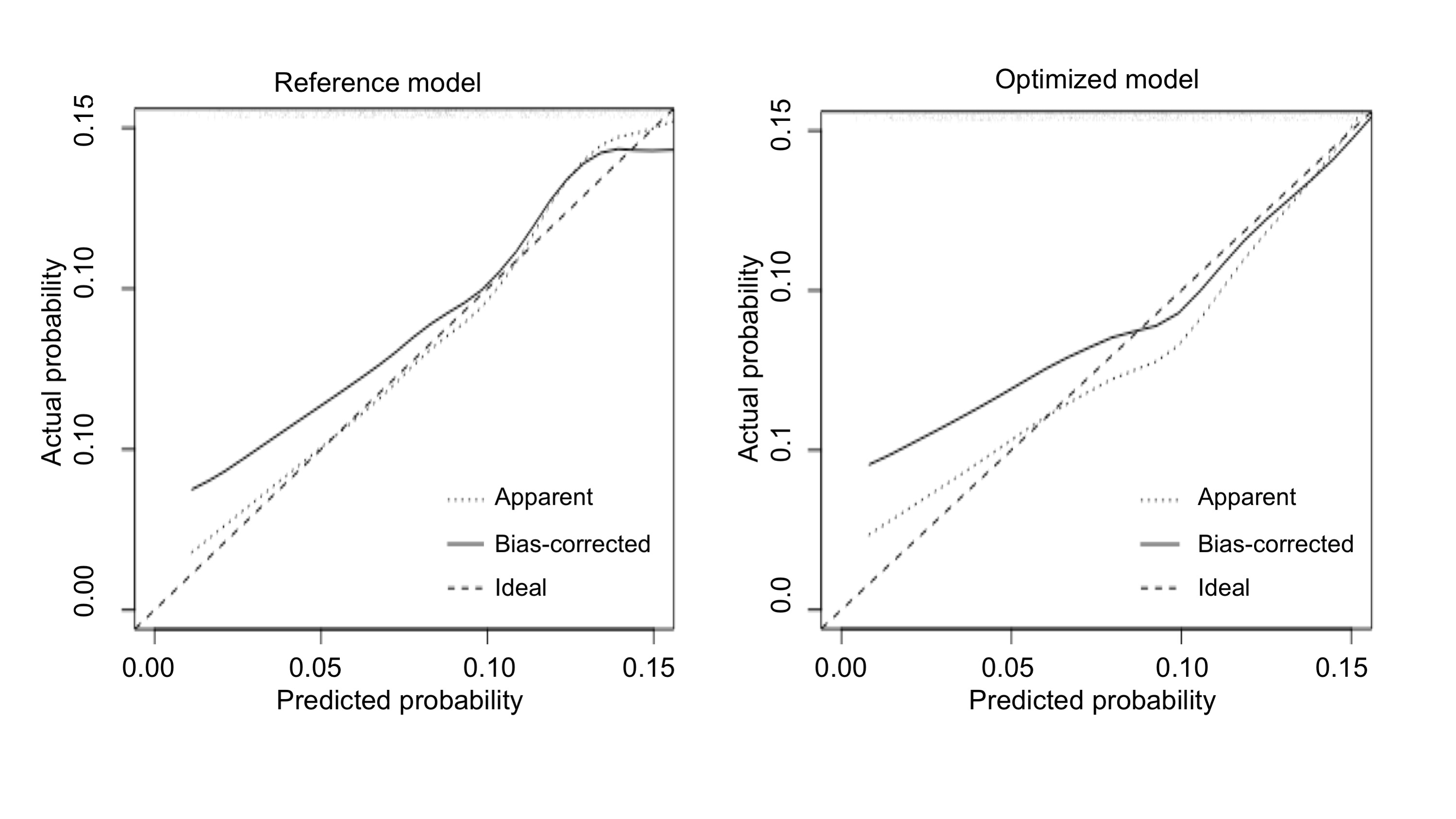
**

**Figure S4- Prediction performance on late readmissions during 8-30 days after hospitalization for COPD in the test set, including patients who readmitted within 7 days after hospitalization for COPD**

**
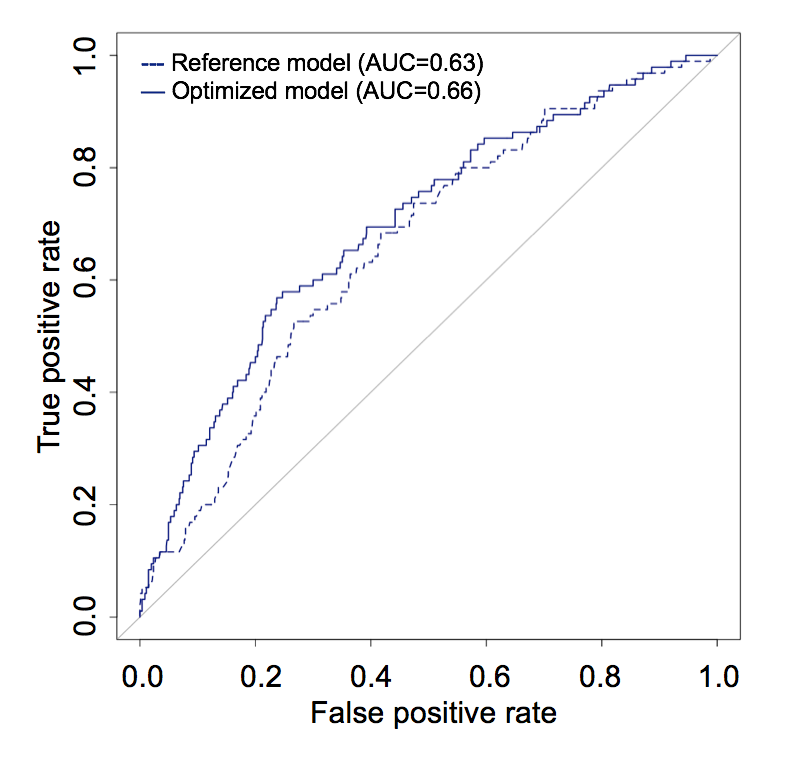
**

**Figures S5. Decision curve analysis for readmission within 30 days after hospitalization for chronic obstructive pulmonary disease**

**A. Overall readmission**

**
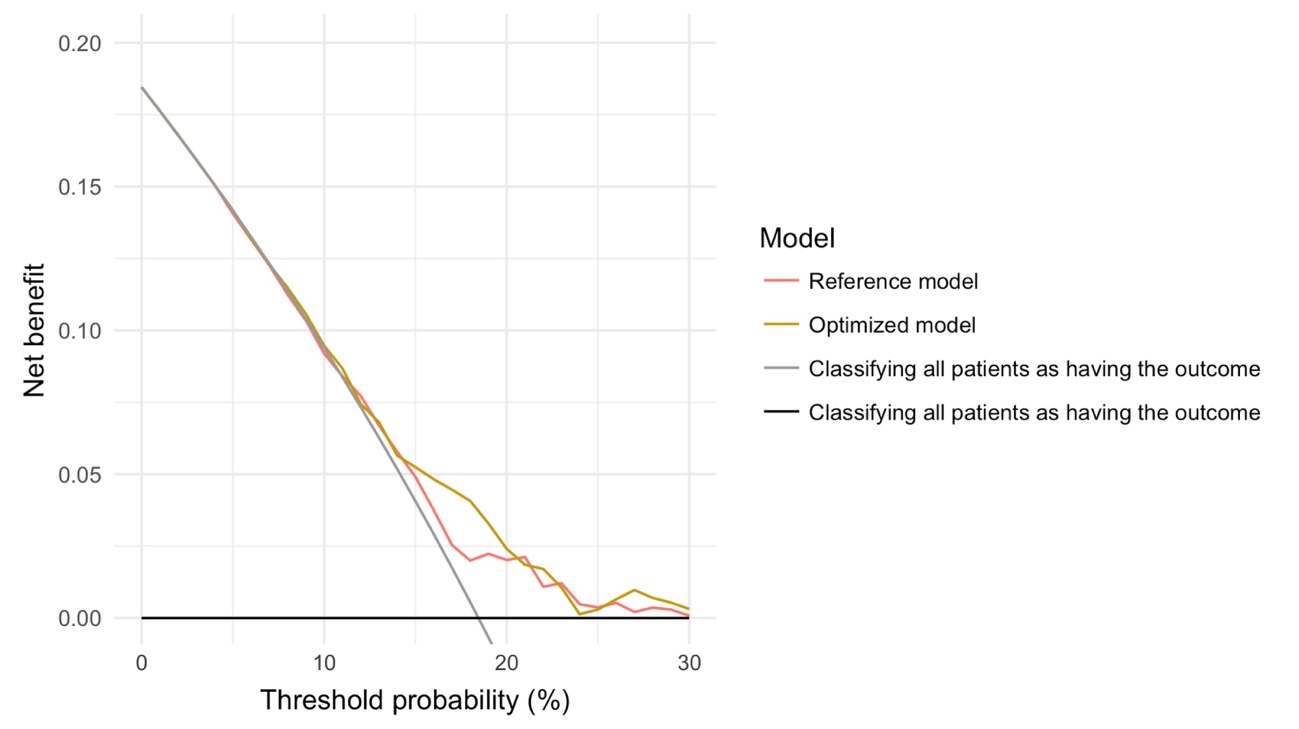
**

**B. Early readmission**

**
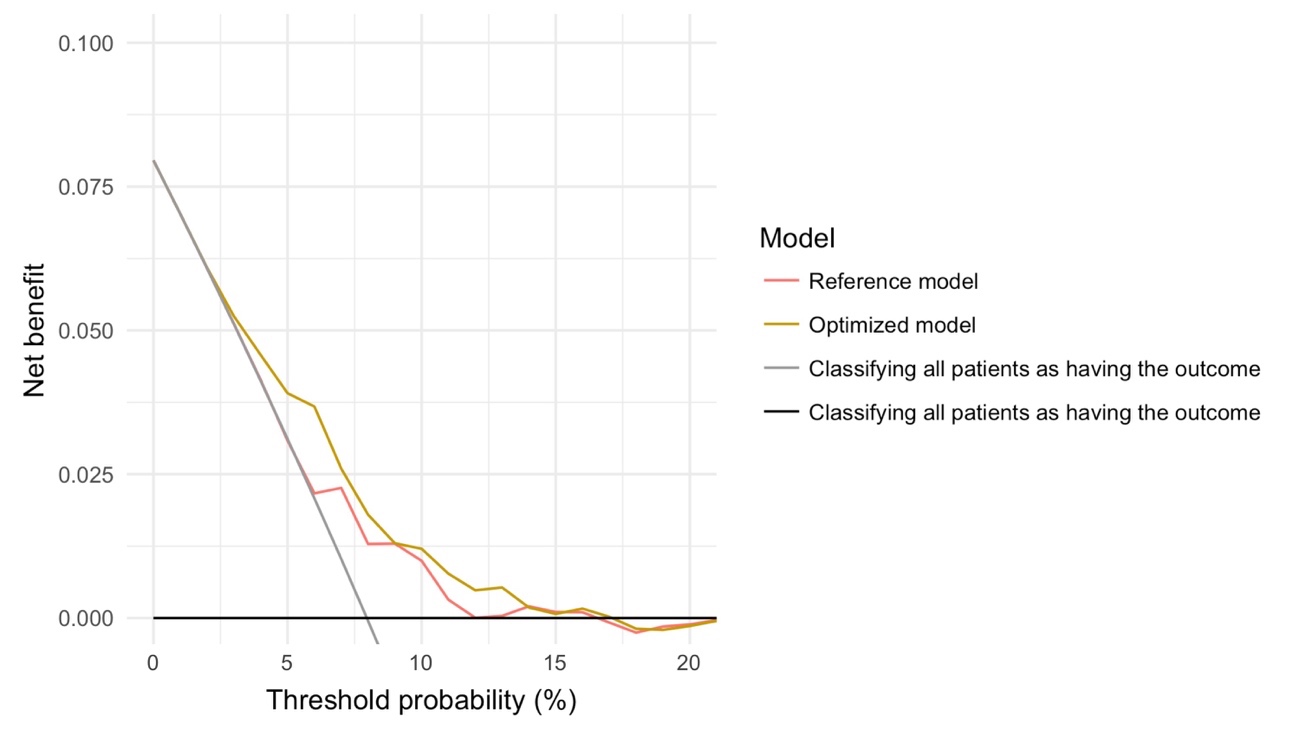
**

**C. Late readmission**

**
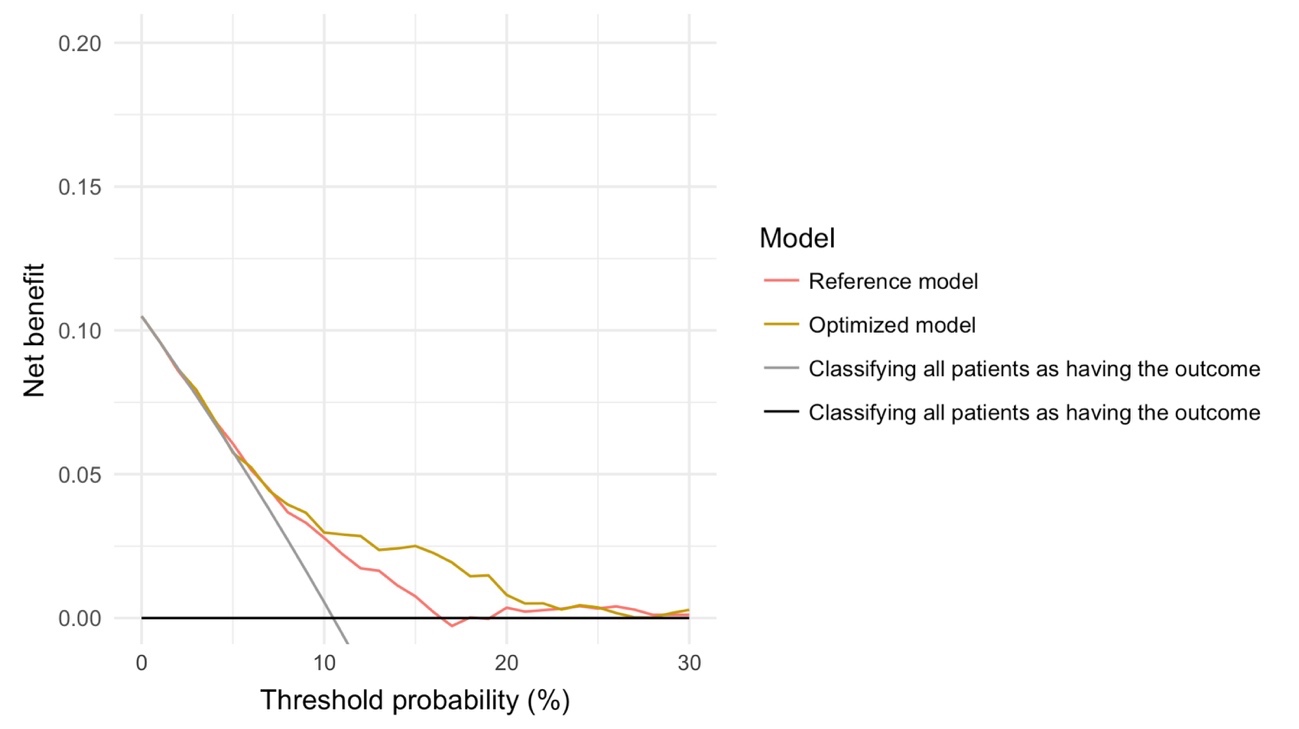
**

**Table S1.** **COPD definitions and corresponding *International Classification of Diseases, Ninth Revision, Clinical Modification* code**

|  | ***ICD-9-CM* code** |
| --- | --- |
| **Primary diagnosis of COPD** |  |
| Obstructive chronic bronchitis with (acute) exacerbation | 491.21 |
| Obstructive chronic bronchitis with acute bronchitis | 491.22 |
| Other chronic bronchitis | 491.8 |
| Unspecified chronic bronchitis | 491.9 |
| Other emphysema | 492.8 |
| Chronic obstructive asthma | 493.2 |
| Chronic obstructive asthma with status asthmatics | 493.21 |
| Chronic obstructive asthma with (acute) exacerbation | 493.22 |
| Chronic obstructive pulmonary disease, unspecified | 496.00 |
| **Primary diagnosis of respiratory failure** | |
| Acute respiratory failure | 518.81 |
| Other pulmonary insufficiency, not elsewhere classified | 518.82 |
| Acute and chronic respiratory failure | 518.84 |
| Respiratory arrest | 799.1 |

Abbreviations: ICD-9-CM, International Classification of Diseases, Ninth Revision, Clinical Modification.

**Table S2.** **Comorbidities included in prediction models and corresponding *International Classification of Diseases, Ninth Revision, Clinical Modification* or *Chronic Condition* codes**

| **Variables** | **Conditions** | **Codes** |
| --- | --- | --- |
| Cardiovascular diseases | Cardio-Respiratory Failure and Shock | CC 79 |
|  | Congestive Heart Failure | CC 80 |
|  | Acute Coronary Syndrome | CC 81-82 |
|  | Chronic Atherosclerosis | CC 83-84 |
|  | Arrhythmias | CC 92-93 |
|  | Other and Unspecified Heart Disease | CC 94 |
|  | Vascular or Circulatory Disease | CC 104-106 |
| Central nervous system diseases | Quadripelgia, Paraplegia, Paralysis, Functional Disability | CC 67-69, 100-102, 177-178 |
|  | Polyneuropathy | CC 71 |
|  | Hypertensive Heart and Renal Disease or Encephalopathy | CC 89 |
|  | Stroke | CC 95-96 |
| Endocrine | Diabetes and DM Complications | CC15-20, 119-120 |
|  | Protein-Calorie Malnutrition | CC 21 |
|  | Disorders of Fluid/Electrolyte/Acid-Base | CC 22-23 |
|  | Other Endocrine/Metabolic/Nutritional Disorders | CC 24 |
| Gastrointestinal diseases | Pancreatic Disease | CC 32 |
|  | Peptic Ulcer, Hemorrhage, Other Specified Gastrointestinal Disorders Other Gastrointestinal Disorders | CC 34 |
| Hematologic diseases | Severe Hematological Disorders | CC 44 |
|  | Iron Deficiency and Other/Unspecified Anemia and Blood Disease | CC 49-50 |
| Musculoskeletal diseases | Decubitus Ulcer or Chronic Skin Ulcer | CC 148-149 |
|  | Cellulitis, Local Skin Infection | CC 152 |
|  | Vertebral Fractures | CC 157 |
| Neoplasms | Metastatic Cancer and Acute Leukemia | CC 7 |
|  | Lung, Upper Digestive Tract, and Other Severe Cancers | CC 8 |
|  | Lymphatic, Head and Neck, Brain, and Other Major Cancers; Breast, Colorectal and other Cancers and Tumors; Other Respiratory and Heart Neoplasms | CC 9-11 |
|  | Other Digestive and Urinary Neoplasms | CC 12 |
| Psychiatric diseases | Dementia or Senility | CC 47 |
|  | Drug/Alcohol Induced Dependence/Psychosis | CC 51-52 |
|  | Major Psychiatric Disorders | CC 54-56 |
|  | Depression | CC 58 |
|  | Anxiety Disorders | CC 59 |
|  | Other Psychiatric Disorders | CC 60 |
| Respiratory diseases | Sleep Apnea | *ICD-9-CM* diagnosis codes: 327.20, 327.21, 327.23, 327.27, 327.29, 780.51, 780.53, 780.57 |
|  | Respirator Dependence/Respiratory Failure | CC 77-78 |
|  | Fibrosis of Lung and Other Chronic Lung Disorder | CC 109 |
|  | Pneumonia | CC 111-113 |
| Other diseases | History of Infection | CC 1, 3-6 |
|  | Renal Failure | CC 136 |

Abbreviations: ICD-9-CM, International Classification of Diseases, Ninth Revision, Clinical Modification; CC, chronic condition code defined by the Centers for Medicare and Medicaid Services

**Table S3. Five most frequent reasons for readmission after hospitalization for chronic obstructive pulmonary disease**

| Readmission diagnosis | n (%) |
| --- | --- |
| Chronic obstructive pulmonary disease | 36 (22%) |
| Pneumonia | 22 (13%) |
| Congestive heart failure | 19 (11%) |
| Respiratory failure | 19 (11%) |
| Coronary atherosclerosis and other heart diseases | 7 (4%) |

Diagnoses were categorized according to the *Clinical Classification Software*

**Table S4. Prediction performance on late readmissions during 8-30 days after hospitalization for COPD in the test set, including patients who readmitted within 7 days after hospitalization for COPD**

| **Model performance measures** | **Late readmission**  **(8-30 days after discharge)** | |
| --- | --- | --- |
|  | Reference model | Optimized model |
| C statistic (95%CI) | 0.63 (0.58 to 0.69) | 0.66 (0.61 to 0.72) |
| Integrated discrimination improvement (95%CI) | — | 0.019 (0.006 to 0.032) |
| Continuous net reclassification improvement,  (95%CI) | — | 0.294 (0.081 to 0.506) |

Abbreviation: CI, confidence interval

For the model comparisons, the integrated discrimination improvement and net reclassification improvement values of >0 indicate that, compared to the reference model, the optimized model has better discrimination and reclassification performance
